# Multimodality Image Post-processing in Detection of Extratemporal MRI-Negative Cortical Dysplasia

**DOI:** 10.3389/fneur.2018.00450

**Published:** 2018-06-14

**Authors:** Wen-han Hu, Xiu Wang, Li-na Liu, Xiao-qiu Shao, Kai Zhang, Yan-shan Ma, Lin Ai, Jun-ju Li, Jian-guo Zhang

**Affiliations:** ^1^Stereotactic and Functional Neurosurgery Laboratory, Beijing Neurosurgical Institute, Capital Medical University, Beijing, China; ^2^Beijing Key Laboratory of Neurostimulation, Beijing, China; ^3^Department of Neurosurgery, Beijing Tiantan Hospital, Capital Medical University, Beijing, China; ^4^Department of Pathology, Peking University First Hospital Fengtai Hospital, Beijing, China; ^5^Department of Neurology, Beijing Tiantan Hospital, Capital Medical University, Beijing, China; ^6^Department of Epilepsy Center, Peking University First Hospital Fengtai Hospital, Beijing, China; ^7^Department of Neuroimage, Beijing Tiantan Hospital, Capital Medical University, Beijing, China; ^8^Department of Neurosurgery, Hainan General Hospital, Haikou, China

**Keywords:** MRI negative, focal cortical dysplasia, morphometric analysis program, PET/MRI co-registration, SPM-PET

## Abstract

**Purpose:** To determine the diagnostic value of individual image post-processing techniques in a series of patients who underwent extratemporal operations for histologically proven, MRI-negative focal cortical dysplasia (FCD).

**Methods:** The morphometric analysis program (MAP), PET/MRI co-registration and statistical parametric mapping (SPM) analysis of PET (SPM-PET) techniques were analyzed in 33 consecutive patients. The epileptogenic zone (EZ) assumed by MAP, PET/MRI, and SPM-PET was compared with the location of the FCD lesions determined by stereoelectroencephalography (SEEG) and histopathological study. The detection rate of each modality was statistically compared.

**Results:** Three lesions were simultaneously detected by the three post-processing methods, while two lesions were only MAP positive, and 8 were only PET/MRI positive. The detection rate of MAP, PET/MRI, SPM-PET and the combination of the three modalities was 24.2, 90.9, 57.6, and 97.0%, respectively. Taking the pathological subtype into account, no type I lesions were detected by MAP, and PET/MRI was the most sensitive method for detecting FCD types II and IIA. During a mean follow-up period of 22.94 months, seizure freedom was attained in 26/33 patients (78.8%) after focal corticectomy.

**Conclusions:** MAP, PET/MRI, and SPM-PET provide complementary information for FCD detection, intracranial electrode design, and lesion resection. PET/MRI was particularly useful, with the highest detection rate of extratemporal MRI-negative FCD.

## Introduction

The success of epilepsy surgery depends on the precise localization and complete resection of the epileptogenic zone (EZ). Extratemporal epilepsy accounts for 34% of epilepsy cases (1) and its surgical management is challenging due to difficulties in EZ localizing and subsequent poor seizure outcomes, especially in MRI-negative cases (2, 3). The main pathological substrate underlying MRI-negative extratemporal epilepsy is focal cortical dysplasia (FCD) (4).

Several non-invasive modalities have been developed to detect these MRI-invisible lesions, including interictal positron emission tomography with ^18^F-fluorodeoxyglucose (^18^FDG-PET) (5), ictal single photon emission computed tomography (SPECT) (6) and magnetoencephalography (MEG) (7). Quite a number of studies have reported that image post-processing techniques, including the morphometric analysis program (MAP), PET/MRI co-registration and statistical parametric mapping (SPM) analysis of PET (SPM-PET), could improve the detection of MRI-negative FCD (8–11). MRI and ^18^FDG-PET are the routine neuroimaging modalities for pre-surgical evaluation of epilepsy used in most epilepsy centers. The purpose of the present study was to compare the localization values of individual post-processing modalities and to study whether a multimodal approach can lead to a favorable post-surgical seizure outcome of extratemporal MRI-negative FCD.

## Materials and methods

### Patients

**Figure 1:**
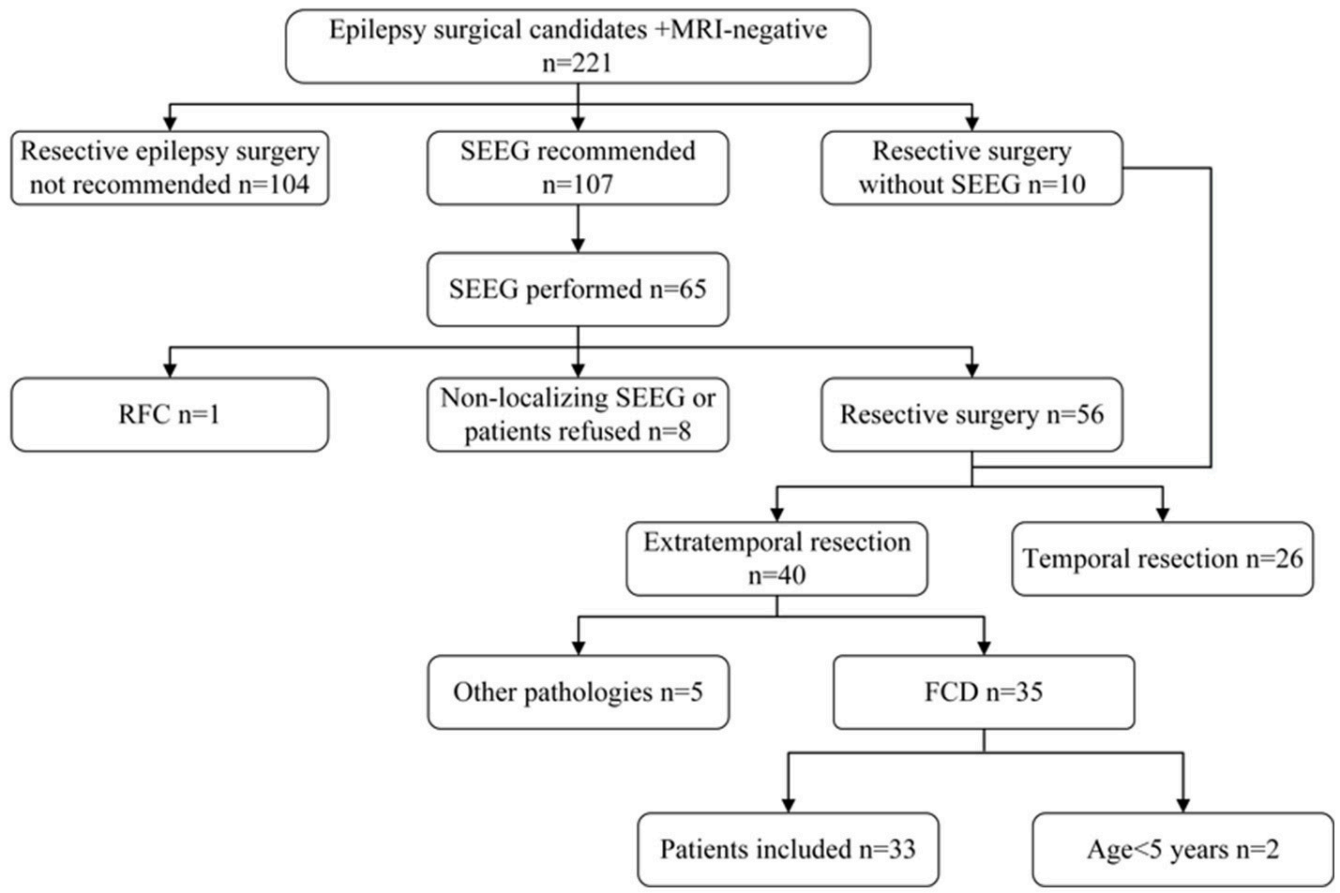
Flow diagram of patient selection. RFC, radiofrequency coagulation.

Among all the patients who underwent a resective epilepsy surgery in our institution between June 2014 and June 2016, we included consecutive patients who met the following criteria: (1) the epileptogenic lesion was undetected by conventional visual analysis on MRI scanning; (2) only extratemporal resection was performed; (3) the pathological finding was FCD; (4) the patient was over 5 years old (12, 13); (5) the post-surgical follow-up was over 12 months and (6) no cranial surgery was performed previously (Figure 1). A detailed pre-surgical evaluation, including medical history, neurological examination, video-EEG monitoring, high-resolution MRI, FDG-PET and image post-processing, was performed for all the patients. Stereoelectroencephalography (SEEG) monitoring was further conducted when non-invasive evaluation findings did not allow a clear definition of EZ. Tailored corticectomies were performed by one neurosurgeon (J.G.Z.). Histopathology was blindly reviewed by a neuropathologist specializing in epilepsy (L.N.L.) according to the ILAE 2011 FCD classification system (14). In brief, FCD type I was defined as abnormal radial and/or tangential cortical lamination, and FCD type II was defined as abnormal cortical lamination with dysmorphic neurons alone (type IIA) or together with balloon cells (type IIB). Thirty-eight healthy subjects with similar age (22.50 ± 3.67 years, male: 17) to the included patients were also recruited to establish a control database of MRI and ^18^FDG-PET scans. The healthy subjects were free from neurological or psychiatric disorders, and cerebral MRI scans were normal. This research was approved by the Institutional Review Boards of the Beijing Tiantan Hospital and informed consent were obtained from all included participants. The study has been performed in accordance with the ethical standards laid down in the 1964 Declaration of Helsinki and its later amendments.

### Image acquisition

The MRI scans were performed on a 3T Siemens Verio scanner (Siemens Medical System, South Iselin, NJ), including a 3D T1 sagittal magnetization prepared rapid gradient echo sequence (MPRAGE, TR/TE 1900/2.53, TI 900, matrix 256 × 256, 1.0 mm thickness), T2 axial (TR/TE 7030/110, matrix 256 × 320, 3 mm thickness), FLAIR axial (TR/TE 8000/94, TI 2371.5, matrix 424 × 512, 3 mm thickness), FLAIR sagittal (TR/TE 8000/96, TI 2371.2, matrix 236 × 256, 3 mm thickness), and FLAIR coronal (TR/TE 8000/96, TI 2371.2, matrix 408 × 512, 3 mm thickness). The ^18^FDG-PET examination was performed using a GE Discovery ST PET-CT system (300 mm FOV, matrix 192 × 192, 3.27 mm slice thickness). An IV injection of ^18^FDG at a mean dose of 220 MBq/70 kg body weight was administered. Reconstructed images were corrected for attenuation using transmission scans obtained from a germanium source. No patients had an ictal event <6 h before or during the PET scan.

### Visual analysis

Before post-processing, conventional visual analyses of the MRI and PET images were independently performed by an epileptologist (X.Q.S.) and a neuroimaging expert (L.A.). Any discrepancies were resolved through discussion. The two reviewers defined MRI-negative as a normal MRI image or one showing nonspecific abnormalities (15). PET visual analysis was a semi-quantitative procedure, a functional tool implanted in GE AW 4.6 workstation was used to calculate the regional maximal value of standardized uptake value (SUV) through visual inspection. The asymmetry >15% with the contralateral homologous regions was considered significant (16).

Clinical data, including scalp EEG and semiology, were also provided during the conventional visual and post-processing analyses. The reviewer assigned to each specific post-processing analysis was blinded to the results of the other analyses.

### MAP processing

MAP processing is an SPM5-based image processing method that compares individual brain MRI scans voxel by voxel with a normal database; the details of this method were described in previous publications (10, 17). Junction and extension images were derived from a 3D T1 image after automatic calculation, which highlights FCD features including blurring of the gray-white matter junction and abnormal deep sulci, respectively. As reported previously, an epileptologist reviewer (K.Z.), blinded to the conventional visual results, used a z-score threshold of 4 (junction image) and 6 (extension image) to identify candidate MAP-positive (MAP+) region (13). The co-registered structural MRI was visually inspected at the candidate MAP+ region. The patient was classified as MAP+ if the structural scan was also considered to be abnormal at this location (11, 13).

### PET/MRI co-registration

The PET images were co-registered to the corresponding 3D T1 image using co-registration algorithms in SPM5, and then, the co-registered PET image was overlaid on the 3D T1 image in MRICron (http://people.cas.sc.edu/rorden/mricron/index.html) with the spectrum displayed and 60–80% transparency. An epileptologist (W.H.H.) reviewed all the fusion images and the asymmetry threshold was set as 15% as did in PET visual analysis. Only the hypometabolic region having concordance with the electroclinical data was classified as PET/MRI-positive (PET/MRI+).

### SPM-PET processing

SPM-PET processing is also an SPM5-based method that converts the individual cerebral PET image to a standardized uptake value ratio (SUVR) image (18) and compares it voxel by voxel with a normal database of 38 healthy subjects by using a two-sample *t*-test. According to previous literature (19), a reviewer (X.W.) used an uncorrected *p*-value of *P* < 0.001 and an extended voxel size (k) threshold of *k* > 200 voxels corrected, to identify candidate SPM-PET positive (SPM-PET+) region. Similar to the PET/MRI analysis, only regions concordant with the electroclinical data were classified as SPM-PET+.

### Validation of post-processing analyses

The EZs assumed by the MAP, PET/MRI, and SPM-PET were compared with the locations of the FCD lesions determined by the SEEG and the pathological examinations. In patients with SEEG implantation into the cortical areas detected by the post-processing techniques, concordance was considered if SEEG contacts within these areas present earliest seizure onset pattern. Otherwise, concordance was considered if more than half of the detected area and the voxels with greatest abnormalities (highest z score for MAP, greatest asymmetry for PET/MRI, and highest *t*-value for SPM-PET) within this area were included in the resection extent.

### Statistical analysis

The differences in the detection rates between the abovementioned diagnostic modalities were compared using Fisher's exact test (two-tailed). Significance was defined as *P* ≤ 0.05. The statistical tests were performed with SPSS 22.0 (IBM Inc., New York, USA).

## Results

### General information

Thirty-three patients (9 female and 24 male) were included and the mean age at surgery was 20.21 ± 8.34 (SD) years old. Twenty-nine patients underwent SEEG monitoring before surgery. Two hundred and twenty-three depth electrodes were totally implanted and 7.69 electrodes were averagely implanted per patient (ranging from 4 to 16 electrodes). Four patients who met the criteria proposed by Chassoux et al. underwent the operation without invasive EEG monitoring (20). The FCD were located in frontal lobe in 25 patients, insular lobe in 6 patients, frontoparietal lobe in 1 patient and occipital lobe in 1 patient. According to FCD subtypes, 5 patients were classified as FCD type I (15%), 28 (85%) as FCD type II (21 FCD type IIA and 7 FCD type IIB). At a mean postoperative follow-up of 22.94 ± 4.19 months, 26 patients (78.8%) became seizure-free (Engel Ia) according to Engel's classification (21). Clinical characteristics, neuroimaging findings and surgical outcomes of the 33 patients are provided in the Supplementary Material (Table e1).

### Visual PET analysis

Initially, the conventional visual analysis identified 19 hypometabolic regions in 14 patients, and a single hypometabolic region was presumed to be the EZ in these patients after the electroclinical data review. Of these 14 patients, 4 had presumed EZs that were nonconcordant with the locations of the histopathologically confirmed FCDs; thus, the visual analysis localized the lesion correctly in 10 patients (type I, *n* = 2; type IIA, *n* = 5; type IIB, *n* = 3), which led to a positive detection rate of 30.3%.

### MAP analysis

**Figure 2:**
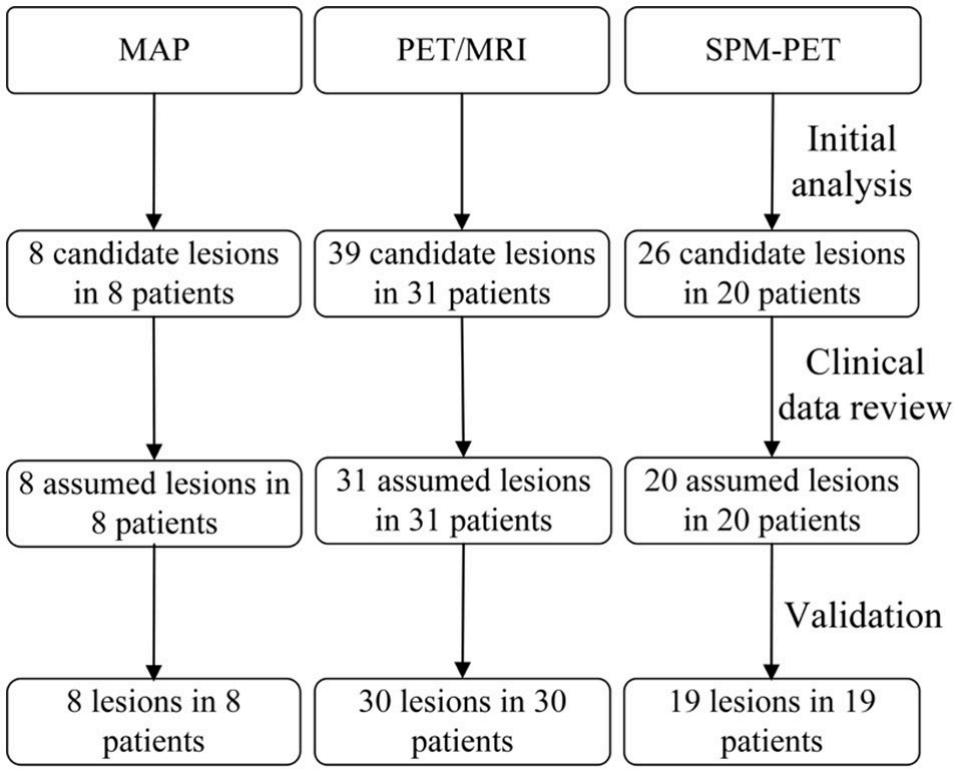
Flow diagram of detection process. The second row refers to the primary analysis results by post-processing methods. The third row refers to the presumed EZ when clinicians read the results with other evaluation information and the last row are the results validated by SEEG or histopathology.

**Figure 3:**
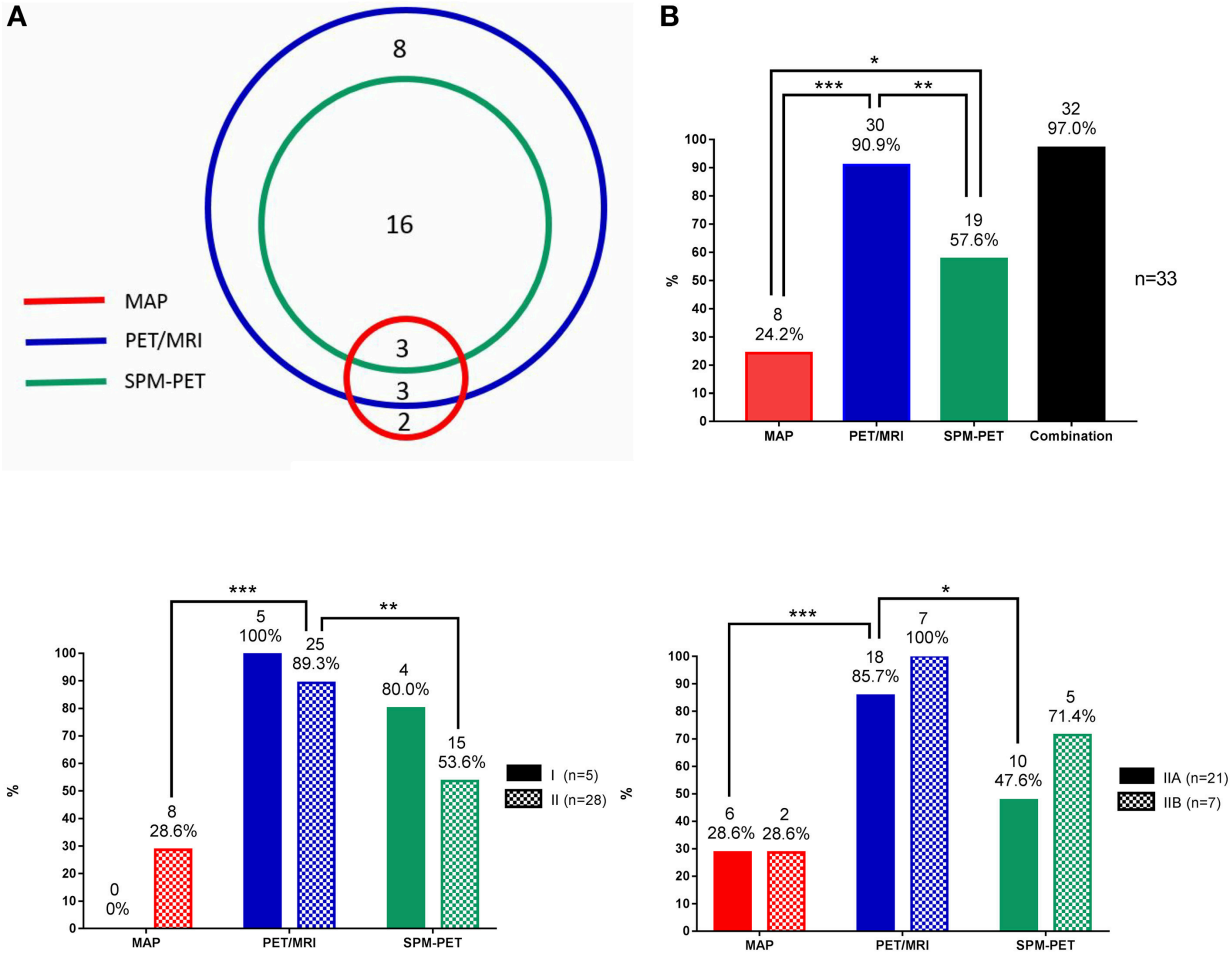
Statistical data of MAP, PET/MRI, and SPM-PET in detecting FCD. **(A)** Number of patients with FCD lesions detected by PET visual analysis, MAP, PET/MRI, and SPM-PET and their concordance. **(B)** Detection rates of MAP, PET/MRI, SPM-PET, and the combination of those methods for all FCD types. **(C)** Detection rates of MAP, PET/MRI, and SPM-PET for FCD types I and II. **(D)** Detection rates of MAP, PET/MRI, and SPM-PET for FCD types IIA and IIB. *P < 0.05; **P < 0.01; ***P < 0.001.

The extension images of all the patients were negative. Eight patients had positive junction images with a single presumed lesion, and all the detected lesions were in agreement with the electroclinical data and histopathological results (Figure 2). When referring to the pathological lesion subtype, six were type IIA and two were IIB, while no type I lesions were detected by this method. Overall, the detection rate of the MAP procedure was 24.2% (Figure 3B).

### PET/MRI analysis

Thirty-nine hypometabolic regions were identified in 31 patients from the PET/MRI fusion images, and 8 regions were excluded due to disagreement with the semiological and/or scalp EEG data. The SEEG and histopathological results revealed that the PET/MRI method successfully detected FCD lesions in 30 patients (type I, *n* = 5; type IIA, *n* = 18; type IIB, *n* = 7) (Figure 2), indicating that the detection rate was 90.9% (Figure 3B). When compared with the conventional visual analysis, the PET/MRI method showed an improved detection rate (90.9 vs. 30.3%, *p* < 0.001).

### SPM-PET analysis

A single hypometabolic region with electroclinical concordance was detected in 20 patients by this quantitative approach. Among these patients, one had an FCD lesion that was nonconcordant with the location of the detected region. Lesions in 19 patients (type I, *n* = 4; type IIA, *n* = 10; type IIB, *n* = 5) were identified correctly (Figure 2), leading to a detection rate of 57.6% (Figure 3B). The detection rate of the SPM-PET method was statistically higher than that of the visual analysis method (57.6 vs. 30.3%, *p* = 0.046).

### Comparison of MAP, PET/MRI, and SPM-PET

**Figure 4:**
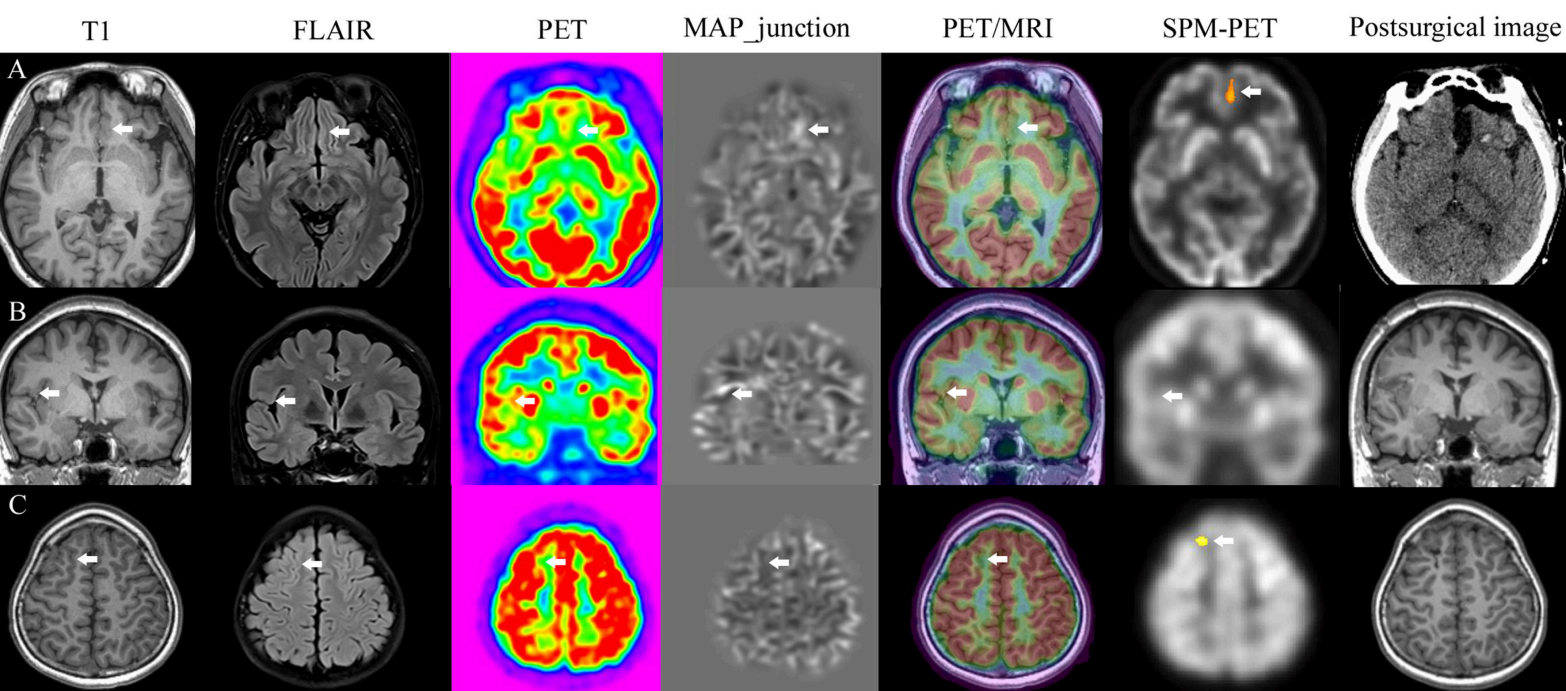
Image post-processing examples. Patients with FCD lesions detected by all methods **(A)**, only MAP **(B)**, PET/MRI, and SPM-PET **(C)**.

**Figure 5:**
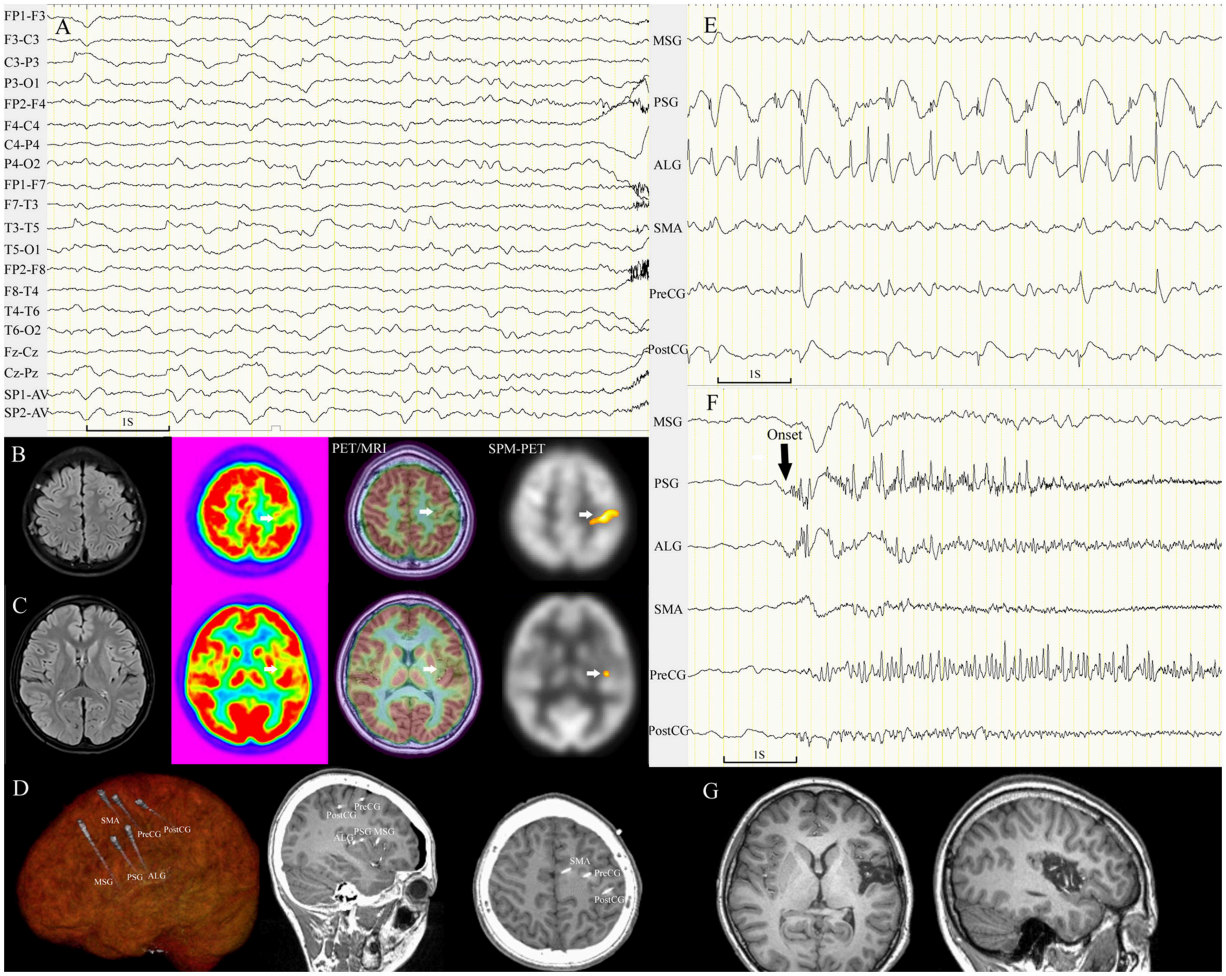
Patient 21 with FCD tended to be mislocalized by conventional visual analysis of PET images. The semiology of the 13-year-old girl was nocturnal dystonic posturing of the left limbs, numbness in the left arm with ambiguous localization before rare diurnal seizures. Scalp EEG showed periodic sharp-slow wave complexes on channel C3-P3 and T3-T5 before seizure onset **(A)**. MRI scans were negative. Focal hypometabolism in the left parietal lobe was identified by conventional visual analysis **(B)**, which was nonconcordant with the ambiguous localization of the numbness and epileptic waves on channel T3-T5. PET/MRI and SPM-PET demonstrated mild hypometabolism in the central sulcus of the left insula **(C)**. Intracranial electrodes were implanted for coverage of the left insular and central areas **(D)**. Interictal SEEG recording showed that continuous, repetitive spikes arose from the left PSG and ALG and spread to the central area **(E)**. Ictal data showing seizures (10 onsets during SEEG monitoring) originating from the left PSG **(F)**. The left PSG, ALG, and PLG were removed **(G)**; the pathological finding was FCD IIA, and the patient was seizure-free through the last follow-up at 21 months. ALG, anterior long gyrus; PLG, posterior long gyrus; PostCG, postcentral gyrus; PreCG, precentral gyrus; PSG, posterior short gyrus.

**Figure 6:**
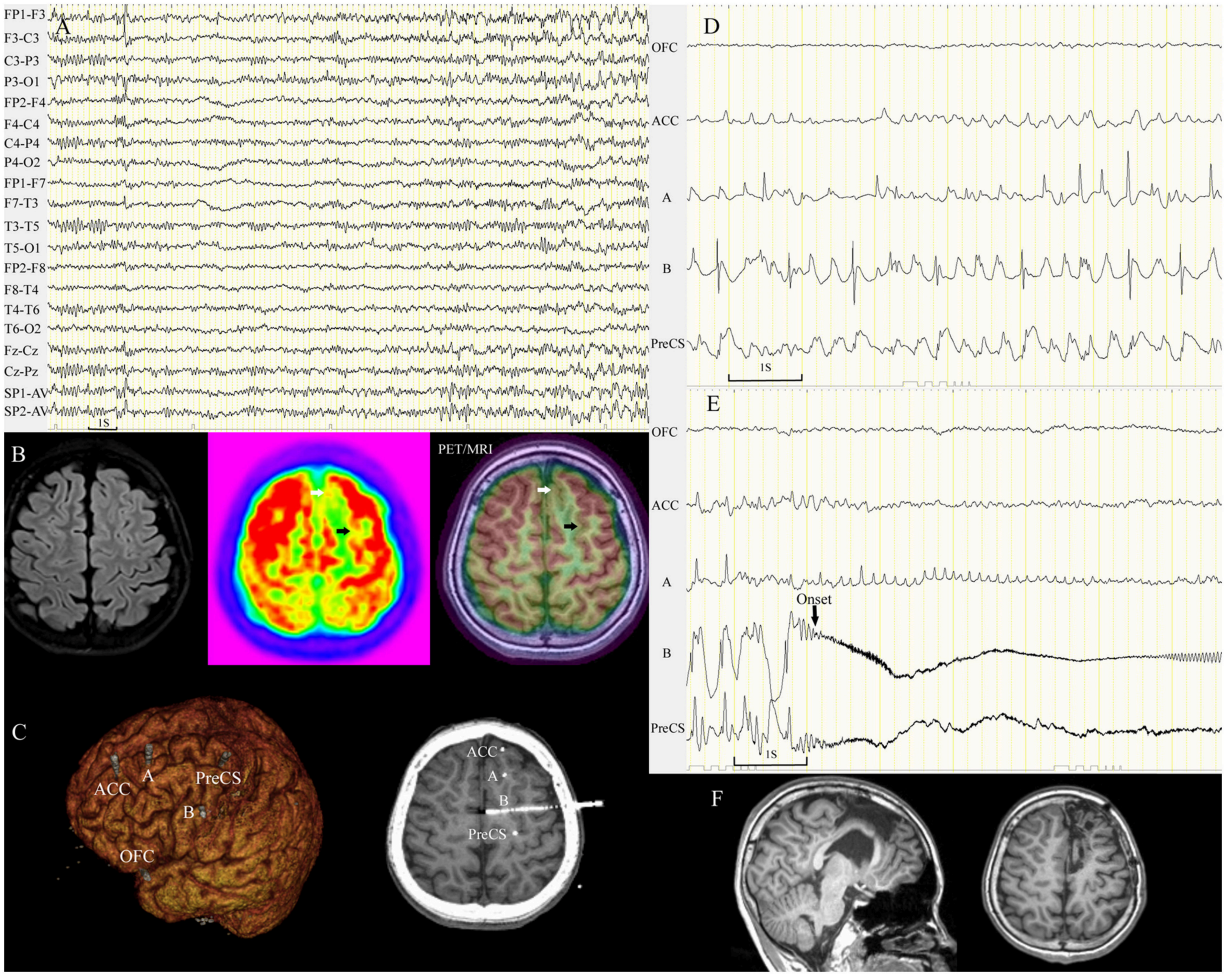
Subtle hypometabolic change tended to be overlooked by conventional visual analysis. A 12-year-old boy (patient 32) with nocturnal seizures manifesting as aura (fear/fluster), tachycardia, fearful expression, dystonic posturing of the right limbs, and hypermotor activity. Ictal scalp EEG demonstrated sharp waves in the left frontal lobe **(A)**. Visual analysis identified focal PET hypometabolism in the left mesial frontal region (white arrow). PET/MRI detected a very subtle hypometabolic change (black arrow) in the ipsilateral superior frontal sulcus **(B)**. Five electrodes were implanted in the left frontal area including the two hypometabolic sulci **(C)**. Asynchronous spikes arose from the two sulci during the interictal period **(D)**, and seizures (14 onsets during SEEG monitoring) originated from the left superior frontal sulcus **(E)**. The two sulci were removed, and both were FCD IIB **(F)**. The patient remained seizure-free through the last follow-up at 18 months. ACC, anterior cingulate cortex; OFC, orbitofrontal cortex; PreCS, precentral sulcus.

As shown in Figure 3A, three lesions were simultaneously detected by the three post-processing methods, two lesions were only MAP+, and 8 lesions were only PET/MRI+. Image post-processing examples are shown in Figure 4. When comparing the detection rates of the three post-processing methods (Figure 3B), PET/MRI had the highest one (PET/MRI vs. MAP, 90.9 vs. 24.2%, *p* < 0.001; PET/MRI vs. SPM-PET, 90.9 vs. 57.6%, *p* = 0.004), followed by SPM-PET (SPM-PET vs. MAP, 57.6 vs. 24.2%, *p* = 0.012). Two illustrative cases showing the values of the PET/MRI and SPM-PET methods are described in Figures 5, 6. Taking the pathological subtypes into account, the PET/MRI method was the most sensitive in detecting FCD types II (PET/MRI vs. MAP, 89.3 vs. 28.6%, *p* < 0.001; PET/MRI vs. SPM-PET, 89.3 vs. 53.6%, *p* = 0.007) (Figure 3C) and IIA (PET/MRI vs. MAP, 85.7 vs. 28.6%, *p* < 0.001; PET/MRI vs. SPM-PET, 85.7 vs. 47.6%, *p* = 0.02) (Figure 3D).

## Discussion

In the present study, we used image post-processing methods to derive additional localizing information from MRI and PET that could be neglected or even invisible with conventional visual analysis. Then, together with comprehensive epilepsy evaluation information, we further compared the complementary value in extratemporal MRI-negative FCD detection provided respectively by MAP, SPM-PET, and PET/MRI co-registration and the findings showed that PET/MRI co-registration provides the most effective complementary information in FCD detection (91%) and the combination of 3 modalities could reach 97%.

A favorable post-surgical seizure outcome, with 78.8% of the patients being seizure-free, was achieved in our series. Improved detection sensitivity of FCD with post-processing methods, especially for subtle or focalized FCD, may contribute to the better outcome than previous similar studies with conventional pre-surgical evaluation (30–46%) (3, 22, 23). Image post-processing techniques have also shown a promising ability to delineate the spatial extent of an FCD lesion (17, 20) or epileptogenic zone in MRI negative cases, like focal hypometabolism in PET/MRI coregistration (8). Based on the data of the multimodality image post-processing and SEEG monitoring, all the patients in our study underwent tailored corticectomies. In patients with subtle lesions, very focal resection, including only one single sulcus or gyrus, was performed.

The extension image of MAP is designed to detect abnormal distribution of the gray matter, and it is more sensitive for heterotopia or schizencephaly than for FCD according to our experience. Therefore, it is not difficult to explain why all the patients in our study showed negative results in the extension image. Only five patients with type I lesions were included in our series, and all of those cases were undetected by MAP. This result has disagreement with a previous report stating that 18 of 28 MAP+ FCD lesions were type I (13). More studies should be carried out to determine the diagnostic yield of MAP for type I FCD lesions. Krsek et al. demonstrated that the most typical MRI finding in FCD type I was a regional reduction of the white matter volume, followed by FLAIR and T2w white matter signal changes (24). Protocols specifically designed to characterize these abnormal changes may be helpful in improving the detection rate of type I lesions. Generally, MAP guides a second reading of structural MRI to identify subtle lesions that tend to be overlooked by conventional visual analysis (11, 13). From this angle, MAP may be insensitive for lesions that are strictly normal on structural MRI scans.

PET/MRI co-registration can improve the sensitivity of PET in detecting FCD (8, 25), especially in lesions with subtle hypometabolic changes. The main principle underlying this is that the fusion image provides a gray matter background. Since the glucose uptake of white matter is lower than that of gray matter, with a single PET image we do not know whether the region with low glucose uptake corresponds to gray matter or not. In the present study, in addition to the 10 lesions localized by conventional visual analysis of PET, 20 additional lesions were identified by the PET/MRI fusion image. Most of these lesions showed very focal hypometabolism within a single sulcus or even the bottom of a sulcus. Two critical issues should be carefully addressed before drawing a diagnostic conclusion from a PET/MRI fusion image. The first is to determine whether the reduction of glucose uptake is physiological or pathological, since glucose uptake in central areas, the temporal pole, and the inferior insula is symmetrically lower than that in other areas in healthy subjects. The second is that the hypometabolic area does not exactly represent the EZ (26) and is sometimes even spatially far away from the EZ (Figure 5). Therefore, in clinical practice, using a PET/MRI fusion image to localize an FCD lesion should involve referring to the clinical data, including semiology and EEG results (27). Moreover, not all dysplastic lesions showed hypometabolic changes during interictal scanning; there were 3 cases with lesions that were not detected by either PET or the subsequent PET/MRI. Bansal et al. identified interictal focal PET hypermetabolism in 33 (6.6%) of 498 children with refractory epilepsy, and the conclusion was made that PET hypermetabolism is associated with a high spike frequency on a scalp EEG and can occur in the absence of ictal events during the peri-injection period (28). However, no hypermetabolic case was observed in our series, which was concordant with the study by Chassoux et al. (8).

To alleviate the disturbance of the metabolic inhomogeneity of different brain regions and the subjectivity of rater judgments in PET analysis, the SPM-PET method was developed to calculate the objective metabolic changes by comparing global cerebral glucose uptake of an individual case to a normal database. The optimal criterion to define the potential epileptogenic zone with SPM-PET has yet to be established. The increased sensitivity of SPM appeared to be offset by decreased specificity (29). An uncorrected *p*-value of *p* < 0.001 was the most common statistical thresholds used in the previous studies (19, 30, 31) and our routine clinic work. The threshold with smaller size will decrease the detection specificity of SPM-PET and relative higher minimum cluster size of 200 voxels were used in our clinic work. According to Mayoral's study, no difference of correct localization rate was found between threshold with *p* < 0.001 with *k* > 100 and threshold with *p* < 0.005 with *k* > 200 (29). Besides, the *t*-value has a reciprocal relationship with the standard deviation of the reference database in a *t*-test. We found that the standard deviations of glucose uptake were large at the sulcus bottom, which led to small *t*-values in those regions. As Besson et al. reported that small FCD lesions are located at the bottom of a deep sulcus (32), SPM-PET may be insensitive to detect a mild hypometabolic changes in those lesions. Nevertheless, SPM-PET provides objective and quantitative information and can be used as a confirmation tool for questionable abnormalities observed in conventional visual or PET/MRI analyses.

Brain MRI and PET scans contain massive amounts of information, requiring intensive exploration to identify the structural and functional changes associated with the EZ. MAP is more specific to detect structural alterations of FCD, but MAP has limited detection sensitivity in subtle FCD cases with normal MRI scans. PET post-processing methods, including SPM-PET and PET/MRI co-registration, are designed only for examining hypometabolic areas, not specific for FCD lesions. Thus, false positivity is more possibly occurred in PET post-processing methods. The results of SPM-PET and PET/MRI co-registration should be interpreted with caution and reviewed with comprehensive clinical information. The visualization of these abnormalities by image post-processing techniques is helpful in intracranial electrode design, determining a subsequent surgical plan and improving the efficiency of pre-surgical evaluation.

According to the present study, PET/MRI co-registration was proved to be the most effective complementary tool in FCD detection among these three post-processing techniques and we therefore recommend its systematic use in pre-surgical workup for epilepsy patients as suggested in previous study (8). The additional advantages of the PET/MRI co-registration technique include: (1) it does not require normal data and is applicable at any epilepsy center; (2) PET/MRI co-registration was obtained by overloading PET images on individual MRI, allowing second reading of MRI data to confirm the subtle structural abnormality; (3) PET/MRI co-registration is a more efficient process and saving time in clinical practices. PET/MRI co-registration was proved to be more sensitive than SPM-PET in FCD detection and SPM-PET provided no additional value after PET/MRI co-registration. Because of normalization onto the template, SPM-PET results could not directly guide the second reading of the individual MRI data. PET data of normal control is also much more difficult to collect. In the present data, MAP detected two additional lesions which were too subtle to be detected by PET/MRI co-registration. Thus, MRI quantitative methods may provide some complementary information for PET/MRI co-registration results. However, on the other hand, we believe quantitative methods like SPM-PET and MAP will also have a promising future in the intelligent diagnosis and machine learning.

In the present study, each image post-processing technique was independently applied as complementary method together with whole clinical information during the pre-surgical evaluations. These three techniques are routinely used in our epilepsy center and frequently reported in previous studies, which does not mean they are the most effective tools in the pre-surgical evaluation. Other important techniques, like automated online quantification method (Scenium software) (33) and three-dimensional stereotactic surface Projection (3D-SSP) for PET quantitative analysis (34) as well as more advanced multimodal lesion profiling analysis of MRI imaging (35), also offer opportunities to optimize the diagnosis and treatment of FCD related epilepsy.

## Limitations

The main limitation of the present study is the absence of inter-rater agreement assessment in the data analysis and we could not exclude the potential bias among reviewers. Our study has several other limitations, including the small number of patients with MRI negative extratemporal FCD. Since electro-clinical data was taken into account during detection procedure, it is impossible to evaluate the specificity of each modality using subjects with normal clinical and EEG data. The threshold of SPM-PET is set based on previous reports and our work experience, we could not rule out the possibility of missing very small focalized FCD. There was a bias of sex distribution between included patients and normal controls. In the whole dataset of epilepsy patients treated in our center, the ratio between male and female was about 1:1 and we collected normal data according to this ratio. The normal database collection was performed before the design of the present study. Although sex was not a factor in the inclusion criteria for the present study, male patients dominated over female patients after patient selection.

## Author contributions

JZ conceptualization of the study and surgery resection. WH design of the study, interpretation of imaging data, statistical analysis and drafted manuscript. XW interpretation of imaging data and drafted manuscript. LL interpretation of FCD pathology data and drafted manuscript. XS interpretation of imaging data and drafted manuscript. KZ interpretation of imaging data and drafted manuscript. LA interpretation of imaging data and drafted manuscript. YM and JL drafted and revised manuscript.

### Conflict of interest statement

The authors declare that the research was conducted in the absence of any commercial or financial relationships that could be construed as a potential conflict of interest.
