# Supplementary material for: Multimodality Image Post-processing in Detection of Extratemporal MRI-Negative Cortical Dysplasia
Source: Front Neurol. 2018 Jun 14;9:450. doi: 10.3389/fneur.2018.00450 (PMC6010529; doi:10.3389/fneur.2018.00450)
Supplement: Supplementary file 1 [file Table_1.DOCX]

Supplementary Material

Multimodality image post-processing in detection of extratemporal MRI-negative cortical dysplasia

Wen-han Hu; Xiu Wang; Li-na Liu; Xiao-qiu Shao; Kai Zhang; Yan-shan Ma; Lin Ai; Jun-ju Li; Jian-guo Zhang

*** Correspondence:** Jian-guo Zhang, email: zjguo73@126.com

Supplement materials

Table e1 Clinical characteristics, neuroimaging findings and surgical outcomes of 33 patients

| Patient | Seizure semiology | Frequency | Ictal scalp EEG | Visual PET^a^ | MAP^a^ | PET/MRI^a^ | SPM-PET^a^ | Num. of electrode | FCD location | Pathological subtype | Follow-up duration, mo | Surgical outcome |
| --- | --- | --- | --- | --- | --- | --- | --- | --- | --- | --- | --- | --- |
| 1 | Head and eyes version to L, hypermotor | daily | R F | **R F** | - | **R F** | **R F** | 6 | R F | IIB | 32 | Ia |
| 2 | Staring, nocturnal hypermotor | daily | L F, C | **L I** | - | **L I** | **L I** | 12 | L I | IIA | 25 | Ia |
| 3 | Uncomfortable feeling in L arm followed by tonic, head and eyes version to L | daily | R F, C, P | - | - | **R F** | - | 4 | R F | IIA | 25 | Ia |
| 4 | Fear and palpitation followed by staring | daily | Not lateralizing | - | - | **L F** | **L F** | 7 | L F | IIA | 25 | III |
| 5 | Fear, tachycardia | daily | Not lateralizing | - | - | **R F** | - | 6 | R F | IIA | 30 | Ia |
| 6 | Head and eyes version to L | daily | R F, C | - | - | **R F** | - | 4 | R F | IIA | 25 | Ia |
| 7 | Fear, tachycardia followed by hypermotor | monthly-daily | R F, T | - | - | **R F** | **R F** | 7 | R F | I | 28 | Ia |
| 8 | Chest stuffiness, flush, numbness in L side of body | monthly | Not lateralizing | - | - | **R I** | **R I** | 9 | R I | IIA | 31 | III |
| 9 | Nocturnal hypermotor | daily | Not lateralizing | - | **R F** | **R F** | **R F** | 8 | R F | IIB | 31 | Ia |
| 10 | Head and eyes turning to L | weekly | R F | **R F**, *T* | - | **R F**, *T* | **R F** | 6 | R F | I | 28 | Ia |
| 11 | Uncomfortable feeling in L side of body followed by dystonic posturing | daily | Not lateralizing | - | - | **R I** | - | 7 | R I | IIA | 24 | Ia |
| 12 | Head turning to R | weekly | L F, T | - | - | **L F** | **L F** | 6 | L F | I | 28 | Ia |
| 13 | Head and eyes version to L | weekly-daily | R F, C | - | **R F** | **R F** | - | 0 | R F | IIB | 21 | Ia |
| 14 | Bilateral eye blinking, head turning to L | daily | Not lateralizing | **L F**, *T* | - | **L F**, *T* | **L F**, *TPO* | 6 | L F | IIA | 21 | Ic |
| 15 | Neck flexion, tonic movement of L limbs | daily | Not lateralizing | - | **R F** | R P | - | 10 | R F | IIA | 22 | Ia |
| 16 | Visual hallucination, staring, L hand automatism | weekly | R O, T, P | **R O**, *T* | - | **R O**, *T* | **R O**, *T* | 9 | R O | IIB | 22 | Ia |
| 17 | Clonic movement of R body | daily | L C, P | - | - | - | - | 16 | L F | IIA | 19 | III |
| 18 | Ictal pouting, head and eyes version to R | daily | Not lateralizing | **L F** | **L F** | **L F** | - | 6 | L F | IIA | 20 | Ia |
| 19 | Uncomfortable feeling in L arm, palpitation followed by GTCS | daily | R F | **R F**, *P* | **R F** | **R F**, *P* | **R F**, *P* | 0 | R F | IIA | 22 | Ia |
| 20 | Numbness in L side of body, head and eyes version to L | daily | R F, C | - | **R F** | - | - | 0 | R F | IIA | 23 | Ia |
| 21 | Numbness in L arm, dystonic posturing of L limbs | daily | L T, C | L P | - | **L I**, *P* | **L I**, *P* | 6 | L I | IIA | 21 | Ia |
| 22 | Numbness in R face, clonic movement of R face | daily | L H | - | - | **L FP** | - | 9 | L FP | IIB | 19 | II |
| 23 | Staring followed by head and eyes version to L | monthly | R F | - | - | **R F** | **R F** | 9 | R F | IIA | 18 | Ia |
| 24 | Bilateral eye blinking, tonic movement of L face | daily | Not lateralizing | L T | - | **R I**, *L T* | **R I** | 13 | R I | IIA | 18 | Ia |
| 25 | Fear and palpitation followed by staring | daily | Not lateralizing | L T | **L F** | **L F**, *T* | **L F**, *T* | 9 | L F | IIA | 20 | Ia |
| 26 | Nocturnal hypermotor | weekly | Not lateralizing | - | - | **R F** | R T | 8 | R F | I | 22 | Ia |
| 27 | Head and eyes version to L, hypermotor | daily | R F | - | - | **R F** | **R F** | 0 | R F | IIB | 21 | Ia |
| 28 | Tonic movement of R face | daily | L F, C, P | L T | - | **L I** | **L I** | 7 | L I | IIA | 18 | Ia |
| 29 | Head and eyes version to L | monthly | R F | **R F**, T | - | **R F**, *T* | **R F**, *T* | 6 | R F | I | 22 | III |
| 30 | Tachypnea, body flexion, Tonic movement of R face, dystonic posturing of R arm | weekly-daily | L H | - | - | **L F** | - | 9 | L F | IIA | 20 | Ia |
| 31 | Fear and palpitation followed by hypermotor | weekly | Not lateralizing | - | - | **L F** | - | 8 | L F | IIA | 19 | Ia |
| 32 | Aura (fear/fluster), tachycardia, fearful expression, dystonic posturing of right limbs, and hypermotor | daily | L F | **L F** | - | **L F** | **L F** | 5 | L F | IIB | 18 | Ia |
| 33 | Numbness in head, ictal pouting, hypermotor | daily | L F | **L F** | **L F** | **L F** | - | 5 | L F | IIA | 19 | II |

Abbreviations: - = negative; C = central; F = frontal; FP = frontoparietal; GTCS = generalized tonic-clonic seizure; I = insular; L = left; O = occipital; P = parietal; R = right; T = temporal; TPO = temporoparietooccipital.

^a^ Results of initial analysis without clinical data review; In italic are the regions excluded after clinical data review; In bold are the detected regions concordant with the epileptogenic zone.
